# Management of Recurrent Ventriculoperitoneal Shunt Infections in Adult Patients

**DOI:** 10.3390/antibiotics14010077

**Published:** 2025-01-13

**Authors:** Neval Elgormus, Yusuf Elgormus, Bagnu Dundar, Fatma Bozkurt, Huseyin Dogu, Hafize Uzun

**Affiliations:** 1 Department of Microbiology, Faculty of Medicine, Istanbul Atlas University, 34303 Istanbul, Turkey; neval.elgormus@atlas.edu.tr; 2 Department of Child Health and Diseases, Medicine Hospital, Istanbul Atlas University, 34408 Istanbul, Turkey; yusuf.elgormus@medicinehospital.com.tr; 3 Department of Medical Biochemistry, Faculty of Medicine, Istanbul Atlas University, 34303 Istanbul, Turkey; bagnu.dundar@atlas.edu.tr; 4 Department of Infectious Diseases and Clinical Microbiology, Faculty of Medicine, Istanbul Atlas University, 34303 Istanbul, Turkey; fatma.bozkurt@atlas.edu.tr; 5 Department of Neurosurgery, Faculty of Medicine, Istanbul Atlas University, 34303 Istanbul, Turkey; huseyin.dogu@atlas.edu.tr

**Keywords:** ventriculoperitoneal shunt, infection, recurrent VPS, resistant Gram-negative bacteria, carbapenem

## Abstract

**Objective:** The objective of this study was to evaluate the demographic, clinical, laboratory, and microbiological features of ventriculoperitoneal shunt (VPS) infections through a 13-year retrospective study. VPS bacterial agents and their antibiotic susceptibility were also investigated through the occurrence of single VPS (SVPS) and recurrent VPS (RVPS) infections. **Methods:** This study included 110 patients with SVPS infections and 55 patients with RVPS infections. **Results:** In patients who developed multiple infections, Gram-negative organisms were found to be the most predominant (60%, 54/90). The resistance rates were 85.2% for third-generation cephalosporins (3GCs), 83.3% for piperacillin–tazobactam, and 10.4% for carbapenem. Of the patients in the SVPS infection group, 49% were treated with combinations with carbapenem; of the patients in the RVPS infection group, 84.4% were treated in the same way. Central nervous system (CNS) tuberculosis as the etiology of hydrocephalus; short duration of antibiotics used for treatment; high cerebrospinal fluid (CSF) protein and blood C-reactive protein (CRP) levels; and prolonged use of prophylactic antibiotics were found to be related to an increased rate of recurrent infection occurrence. A two-stage shunt change approach decreased the risk of recurrent infections. **Conclusions:** Based on the findings of our study, it is essential to closely monitor patients with independent risk factors for RVPS infection development, due to the high rates of resistant Gram-negative bacterial growth and the initiation of empirical antimicrobial treatment with glycopeptide plus carbapenem. Certain treatment options, such as 3GCs plus glycopeptide, should be revised based on clinical progress and microbiological culture results.

## 1. Introduction

Hydrocephalus, i.e., the enlargement of the intraventricular system and subarachnoid space, is a pathological clinical process that may occur as a complication of intracranial hemorrhage, tumor, infection, or brain injury [1]. For the management of this complication, ventriculoperitoneal shunts (VPSs) are used to decrease intracranial pressure. However, the risk of postoperative infection is high, and infection may lead to an unsuccessful outcome and intracranial infection. The management of VPS infection is crucial because of its life-threatening progress [2]. 

The following risk factors for shunt infection have been identified: previous shunt-associated infection, revision of dysfunctional shunts, and postoperative cerebrospinal fluid (CSF) leakage [3,4,5,6,7,8,9]. There are very few studies on the evaluation of risk factors and treatment approaches related to recurrent VPS infection. The non-standard use of antibiotics and inadequate guidelines for the management of VPS infection can lead to an increase in antibiotic resistance and recurrent infections [8,9,10,11].

Research on risk factors in literature usually draws upon different pediatric and adult cases. In pediatric cases, gender, age at shunt insertion (young age; <6 months), preterm birth, immune status, etiology of hydrocephalus, type of hydrocephalus, recent shunt revision, previous shunt infection, opening of the skin at the shunt insertion or incision site, CSF leakage from the incision site, prolonged operation time, surgeon experience, lack of prophylactic perioperative antibiotics, and the use of a neuroendoscope during the operation are the main risk factors [2,12,13,14]. The major risk factors in adult cases are infection of the previous shunt, revision of the shunt for dysfunction, multiple revisions, CSF leakage from the postoperative skin incision, advancing age (advanced age), operation time, the surgeon’s experience, and the use of a neuroendoscope. Age is another factor that increases the risk for shunt infection. Despite advanced surgical techniques, the risk for shunt infection is higher in younger children [2,15].

A longer duration of surgery, less experience of the surgeon, and the priority of the case (emergency) were independent risk factors for shunt complications. Bivariate logistic regression showed that a duration of surgery more than 1 h compared to 1 h or less, an experienced surgeon compared to residents, and an emergency surgery compared to elective surgery were significant risk factors, while emergency surgery was the only significant variable for shunt failure based on multivariate regression analysis [16].

VPS surgery is the primary treatment option for patients with hydrocephalusthroughout the world. Hydrocephalus can be divided into 3 types: congenital, acquired, and normal-pressure hydrocephalus. Disorders that typically require shunting include the following: congenital hydrocephalus after aqueductal stenosis which is a genetic disorder which can cause deformations of the nervous system and is associated with mental retardation, abducted thumbs, and spastic paraplegia; communicating hydrocephalus secondary to meningitis or subarachnoid hemorrhage; myelomeningocele which causes the development of hydrocephalus because the flow of CSF is altered due to hindbrain malformation; craniosynostosis which occurs when the sutures of the skull close too early with sutures fusing before the brain stops growing and in rare occasions can cause hydrocephalus; normal-pressure hydrocephalus which causes a classic triad of memory problems/dementia, gait dysfunction, and urinary incontinence. VPSs are used to treat hydrocephalus and divert CSF from the lateral ventricles into the peritoneum [16]. However, there appears to be no clear agreement among the data that were reported, and the risk factors for VPS infection are still not well known [17]. 

In the present study, we aimed to retrospectively analyze a large series of data from patients who experienced long-term proximal VPS infections; the goal was to describe the epidemiological, clinical, laboratory, and microbiological characteristics and treatment outcomes and to identify the risk factors for recurrent infection in adults.

## 2. Materials and Methods

This case–control study was conducted at the Istanbul Atlas University, Medicine Hospital, Department of Infectious Diseases and Clinical Microbiology, and University of Health Science, Gazi Yaşargil Education and Research Hospital, Department of Infectious Diseases and Clinical Microbiology. This study was conducted according to the guidelines of the Declaration of Helsinki and was approved by the Ethics Committee of the Medical Faculty of Istanbul Atlas University (Number: E-22686390-050.99-26197; Date: 12 April 2023).

Patients who were 12 years old or older and who were diagnosed with VPS infection in a university hospital between May 2006 and May 2019 were included in the present study. This study included 110 patients with SVPS infection and 55 patients with RVPS infection. Patients were divided into 2 groups: single (SVPS) infection with a history of only one infection; recurrent VPS (RVPS) infection with a history of more than one infection episode. Demographic and epidemiological data at the time of VPS infection diagnosis (i.e., age, gender, hydrocephalus etiology, type of hydrocephalus, history of previous shunt intervention, duration of prophylactic antibiotic use, reason of revision surgery, clinical and laboratory findings, and medico-surgical approach) were recorded on the prepared form. 

Standard treatment is a combination of a 3rd generation cephalosporin and glycopeptide, 10–14 days of treatment, and a surgical procedure if indicated. The treatment is revised when necessary, according to the microbiological culture results.

CSF, shunt catheter, shunt trace, wound on the scalp, and intra-abdominal fluid culture results were evaluated. The obtained cultures were identified based on the Clinical and Laboratory Standards Institute (CLSI) criteria. Microorganisms were identified through the Vitek 2 (Biomerieux) automated system, and an antibiogram was performed to determine their sensitivity patterns. Antimicrobial susceptibility testing (AST) with N:420–423 panels for Gram-negative bacteria and Gram-positive bacteria and an AST P664 panel for staphylococci and a st03 panel for streptococci were used. Each VPS infection was defined based on the following criteria:

1. Pathogen growth in the cultures obtained from CSF or shunt equipment;
2. Increase in cells in CSF (>10/mm^3^) and decreased CSF glucose (45 mg/dL) along with the presence of clinical symptoms such as fever, nausea–vomiting, headache, focal neurological deficits, and neck stiffness despite the absence of pathogen growth in obtained cultures [18].

After the routine blood tests and CSF sampling of patients with suspected VPS infection, empirical antibiotic treatment was initiated; then, the surgical procedure was planned. 

In the two-stage shunt replacement process, the infected shunt was removed, and temporary drainage was provided with external ventricular drainage (EVD) until CSF sterilization was achieved. Then, a second shunt was inserted. Surgical procedures to externalize the distal shunt and re-internalize it after ensuring CSF sterilization with antibiotic treatment include the following: (i) the shunt exchange (simultaneously removing the infected shunt and installing a new shunt); (ii) the shunt exchange, removing the infected shunt installing a temporary EVD; and (iii) replacing a new shunt after ensuring CSF sterilization. Empirical antibiotic treatment was revised based on the clinical outcomes and/or culture results. The patients who underwent shunt intervention, shunt revision, and infection at other hospitals and with an infection on the distal end of the shunt were excluded from the present study. 

### Statistical Analyses

Data were analyzed using SPSS 22.0 (SPSS Inc., Chicago, IL, USA) software for Windows. The categorical variables were comparatively analyzed through the χ^2^ test or Fisher exact test, whereas a Student’s *t*-test was employed for the continuous variables. If normal distribution was not achieved, then a Mann–Whitney U test was used. Logistic regression analysis was performed to determine independent risk factors. *p* values lower than 0.05 were considered statistically significant.

## 3. Results

The demographic and epidemiological characteristics for the included patients are summarized in Table 1. Obstructive hydrocephalus and previous shunt intervention histories were found to be more common in the RVPS infection group (*p* < 0.05) than the SVPS infection group. The main indications for VPS surgery were shunt infection in the RVPS infection group and dysfunction of the shunt in the SVPS infection group (*p* < 0.05). In both groups, shunt dysfunction was the indication for shunt surgery. However, the rate was higher in the RVPS infection group (*p* < 0.05). Etiologies of hydrocephalus were central nervous system (CNS) malformation (32.3%), CNS tumor and cyst (23.9%), CNS tuberculosis (23.4%), and intracerebral hemorrhage (20.4) (Table 1). Overall, 34 of 165 patients died (20.6%). Cultures were obtained from 214 CSF samples, 36 samples of shunt equipment, 32 wounds, and 19 intra-abdominal fluid materials.

The diagnosis, clinical characteristics, and laboratory findings for the patients are shown in Table 2. Meningitis episodes occurred at rates of 94.5% and 72%, while ventriculitis episodes occurred at rates of 6.5% and 17% in the SVPS infection and RVPS infection groups, respectively. 

Fever occurred in 44.9% of SVPS infections and 55.1% of RVPS infections; CNS infection occurred in 46.2% of SVPS infections and 53.8% of RVPS infections; intra-abdominal findings occurred in 47.8% of SVPS infections and 52.2% of RVPS infections; local infection occurred in 55.0% of SVPS infections and 45.0 of RVPS infections. CRP and CSF protein were found to be significantly higher in the RVPS infection group than in the SVPS infection group (<0.026 and <0.001, respectively). 

A total of 90 microorganisms grew in 90 episodes in the RVS infection group, while 73 microorganisms grew in 73 episodes in the SVP infection group. Gram-negative bacillus growth (60%) was most common in RVPS infections, while Gram-positive coccal growth (60.3%) was predominant in SVPS infections (*p* < 0.05) (Table 3). In the RVPS infection group, methicillin in staphylococci, third-generation cephalosporin (3GCs), piperacillin–tazobactam, and carbapenem resistance rates in Gram-negatives were statistically higher compared to those in the SVPS infection group (*p* < 0.05) (Table 3). 

While 84% of patients were treated with combinations of carbapenem in the RVPS infection group, this rate was 49% in the SVPI infection group (*p* < 0.05) (Table 4). The duration of antibiotic use was shorter in the RVPS infection group, and the difference was statistically significant (*p* < 0.05) (Table 4). The rate of two-stage shunt change was lowest in the VPS infection group, while it was the highest in the SVPS infection group (*p* < 0.05) (Table 4).

In the group comparison, 10 of the variables were found to be statistically significant (previous shunt insertion, hydrocephalus etiology, type, shunt revision reasons, CSF protein, CRP, duration of prophylactic antibiotic use, growth of microorganisms and duration of antibiotics used for treatment, and two-stage shunt change). When the logistic regression analysis was performed, the presence of prior shunt insertion, CSF protein, and total duration of antibiotics used for treatment were found to be independent risk factors.

Logistic regression analysis was performed to determine independent risk factors (Table 5). In the logistic regression, it was observed that CSF leakage, history of previous shunt infections, CSF protein, lack of 2-stage shunt exchange, and CNS infection were statistically significantly effective in the development of risk factors.

## 4. Discussion

Recurrent VPS infection remains a major problem, and, unfortunately, there are not many studies on this topic. It is challenging to find detailed data on adult patients with VPS infections in the literature [15,19,20,21,22]. In the current study, we examined the risk factors that are present in the development of recurrent infection in adult patients with VPS and its management. This study included patients with single and recurrent infections. We observed that carbapenem has a powerful effect on recurrent infections. Use of long-duration prophylactic antibiotics may increase the risk of recurrent infection. If the causative bacteria are Gram-positive, then antibiotic treatment should be given for 14 days, but if they are Gram-negative, then antibiotic treatment should be given for 21 days. In the cases addressed here, antibiotics were used for less than 21 days despite the Gram-negative growth. We found increased Gram-negative bacteria growth and increased resistance to the antibiotics in the RVPS infection group. Carbapenem sensitivity was higher than the other agents for recurrent infections. In addition, Gram-negative bacteria growth and resistance to 3GCs are risk factors for recurrent infections.

Several studies have reported that certain etiological factors of hydrocephalus are associated with increased rates of VPS infection. In a study which investigated 1015 patients who received VP shunts, 70% of which were adults, congenital hydrocephalus was the most common etiology of hydrocephalus in those with VPS infection; this was followed by hydrocephalus due to cerebral hemorrhage, post-traumatic and post-craniotomy events, and tumors/cysts [7]. In another study examining 290 VPS and RVPS infections in children, congenital malformations took the first place in the etiology of hydrocephalus, followed by CNS tumors and CNS infections [8]. Congenital malformations constituted the most common etiology of hydrocephalus in the SVPS infection group, while CNS tuberculosis was the most common in the RVPS infection group, in accordance with the studies in the literature. Hydrocephalus is one of the most common complications of tuberculous meningitis (TBM) [2,3]. Simon et al. [3] found computer tomography (CT) evidence of hydrocephalus in 83% of 193 children with TBM. In a CT study, only 3 out of 60 children and adults with TBM were found to have normal ventricles, giving an incidence of 95% [6]. It has been reported that complications of shunt surgery are higher in patients with TBM than in patients with other conditions. The reasons for this are the poor general condition of these patients and the presence of higher protein and cellular content in the CSF leading to more frequent shunt obstruction. Sacar et al. [23] reported shunt-related complications in 11 (30%) children and 3 of 37 children that had to undergo multiple shunt revisions. Reddy et al. [7] reported that 26 of 114 (22.8%) patients had to undergo one or more shunt revisions, while 1 patient required more than three revisions. Wang et al. [24] reported a shunt infection rate of 15.6% and revision rate of 43.8% in their series of 37 children who underwent shunt surgery for TBM with hydrocephalus. Multiple revisions were performed in 18.7% of the patients. Shunt infection and erosion of the skin over the shunt components are the other major complications of shunt surgery in poor-grade patients with TBM and hydrocephalus. Mortality on long-term follow-up has been reported to vary from 10.5% to 57.1% [25]. To our knowledge, this is the first study to evaluate CNS tuberculosis and other etiological factors for hydrocephalus in the development of VPS infection. In our RVPS infection group, CNS tuberculosis was the most common etiology of hydrocephalus, and the rate of VPS infection episodes was higher in this etiologic group than in other etiologic groups. Consistent with the above studies, the rate of shunt revision history, mean CSF protein, and mortality rate were high in our RVPS infection group. In addition, high CSF protein was found to be an independent risk factor for the development of RVPS infection. Early diagnosis and treatment of CNS tuberculosis will prevent morbidity and mortality.

Several studies indicated that obstructive hydrocephalus [7] and non-obstructive hydrocephalus [11,15] were risk factors for the development of VPS infection. In the present study, obstructive hydrocephalus was found to be a risk factor in the development of recurrent VPS infection. CSF leakage in children, especially after the closure of meningomyelocele, increases the risk of developing shunt infection [26]. In a prospective study of 205 pediatric cases, it was determined that CSF leakage increased VPS infection development by 27 times [26]. The present study found that CSF infection was a risk factor for the development of RVPS infection in adults. In a study that included pediatric patients, higher CSF protein level prior to VPS insertion was associated with a potential risk of reinfected VPS [8]. It was mentioned that high CSF protein levels were found to be associated with the development of shunt reinfection [27]. Similarly, we observed higher CSF protein levels at the first episode diagnosis in the RVPS infection group when compared with the single-episode group. Higher CRP levels were associated with recurrent infections. In addition, CSF protein was found to be an independent risk factor. Data on protein leakage suggest the presence of a block of the CSF circulation. In this condition, antibiotic concentrations in the site of infection are reduced, and this could be a major cause of treatment failure.

The common treatment recommendation for the prevention of VPS infection is a single dose of cefazolin [28,29]. The present study established that prolonged use of antibiotic prophylaxis was a risk factor in patients with recurrent infections. The use of antibiotics for a long time off-label disrupts the intestinal flora of the patient and may lead to resistant bacterial infections through the colonization of hospital-acquired resistant bacteria. Accordingly, bacteria grown in patients who developed RVPS infection were found to be hospital-borne, resistant bacteria [30].

VPS infection is often caused by Gram-positive bacteria [31]. However, several studies have indicated increased rates of resistance and Gram-negative bacteria in recent years [8,23,24,32]. Studies conducted in Turkey reported that 50% of the grown bacteria were Gram-negative [23,24]. Another study reported that 20 of the 46 grown bacteria belonged to the *Staphylococcus* strain, and 65% of those were oxacillin resistant [24]. In another study, cephalosporin sensitivity to Gram-negative bacteria was determined to be 30 to 40%, and carbapenem sensitivity was found to be more than 60%. Similarly, cephalosporin susceptibility to Gram-positive bacteria decreased over the years, while the susceptibility of teicoplanin and Linezolid was found to be more than 60% [32]. According to our knowledge, the bacteria grown and the resistance states were not analyzed by comparison between single and recurrent infection periods. Similarly, we found increased Gram-negative bacteria growth and increased resistance to the antibiotics in the RVPS infection group. Carbapenem sensitivity was higher than the other agents for recurrent infections. In addition, Gram-negative bacteria growth and resistance to 3GCs are risk factors for recurrent infections. The American guidelines, without a recommendation regarding VPS infections, recommend the use of antibiotics for 10–14 days in the presence of Gram-positive growths and 21 days for Gram-negative growths (for healthcare- and device-associated central nervous system infections). In the current study, Gram-positive growth was prominent in the SVPS infection group, and the duration of antibiotic use was consistent with the guideline recommendation; meanwhile, Gram-negative growths were prominent in the RVPS infection group, and the duration of antibiotic use was below the guideline recommendation. At the same time, a short duration of antibiotic use was found to be an independent risk factor for the development of RVPS infection.

The medico-surgical approach (from the American guidelines and a few studies) recommends the removal of the infected shunt and the placement of a temporary EVD until CSF sterilization and, if necessary, the insertion of a new shunt and simultaneous glycopeptide, plus 3GCs, covering Gram-negative and -positive bacteria [10,11,15,16,17,18,19]. A study that examined 78 VPS infection episodes reported no relapses or reinfections among those who underwent two-step shunt replacement, while recurrent infections were observed in other surgical procedures [15]. Another study that focused on 86 VPS infection episodes indicated that minimum failure occurred with the two-step shunt replacement employed via the medico-surgical approach [11]. Another study, which examined 160 single episodes and 90 reinfections, mainly employed the combined treatment of vancomycin plus ceftriaxone, followed by vancomycin plus carbapenem [8]. Similarly to results reported in the literature, the present study determined that low rates of two-step shunt replacement were a risk factor in RVPS infection development. Because bacteria form a biofilm layer in the infected shunt, even if the infected CSF circulating in the shunt is treated, relapses of infection may occur due to bacteria released from the biofilm layer into the CSF. For this reason, in the two-stage change, the possibility of reinfection decreases as the infected shunt is removed, the CSF is sterilized before the second shunt is inserted, a temporary external ventricular drainage EVD is performed, and then the shunt is inserted. However, in those whose infected shunt is not replaced, recurrence may occur because the biofilm layer remains. In a single-stage change, since the CSF is inserted without sterilization, it can reinfect the new shunt or form a new biofilm layer.

The present study has several limitations. First, the APACHE score, or severity of illness, was not measured. The second limitation was the small sample size, which led to an inability to determine the individual effects of each broad-spectrum antibiotic. Finally, this study was conducted in a single healthcare setting; thus, our findings might not be generalizable to other healthcare environments. 

Single and recurrent VPS infections are some of the most common and important complications of this intervention. Based on the findings of our study, it is essential to closely monitor patients with independent risk factors for RVPS infection development, due to the high rates of resistant Gram-negative bacterial growth and to initiate empirical antimicrobial treatment with glycopeptide plus carbapenem. Approaches such as 3GCs plus glycopeptide (and similar treatment options) should be revised based on clinical progress and microbiological culture results. It was observed that the CSF leakage, history of previous shunt infections, CSF protein, lack of the two-stage changes, and CNS infection had a significant effect in the logistic regression analysis. More comprehensive information is needed to create an effective algorithm for RVPS infection management.

## Figures and Tables

**Table 1:** Demographic and epidemiological characteristics of patients.

	SVPS (n = 110)	RVPS (n = 55)	*p*
**Patient age**	33.4 ± 16.7	32.4 ± 15.9	0.826
**Female/Male**	50/60 (45/55)	57/68 (46/54)	0.136
**Hydrocephalus etiology**			
Intracerebral hemorrhage	16 (14.5)	32 (25.6)	0.036
CNS tumor and cyst	37 (33.6)	19 (15.2)	**0.001**
CNS infections	13 (11.8)	42 (33.6)	**<0.001**
CNS malformation	44 (40)	32 (25.6)	**0.019**
**Hydrocephalus Type**			**0.043**
NPH	15 (13.7)	8 (6.4)	0.062
Obstructive hydrocephalus	65 (59)	92 (73.6)	**0.018**
Communicating hydrocephalus	30 (27.3)	25 (20)	0.189
**Previous shunt insertion history**	34 (30.9)	87 (69.6)	**<0.001**
**Shunt revision history**	79 (71.8)	102 (81.6)	0.075
**Shunt revision reasons**			**0.003**
Shunt infection	4 (3.6)	22 (17.6)	**0.001**
Shunt tip dislocation	12 (10.9)	10 (8)	0.445
Shunt obstruction	18 (16.4)	13 (10.4)	0.178
Dysfunction	31(28.2)	30 (24)	0.446
CSF leakage	14 (12.7)	27 (21.6)	0.074
**Prophylactic antibiotic duration (day)**	2.6 ± 1.5	3.3 ± 2.2	**0.014**
**Hospitalization (day)**	21.4 ± 7.8	28.7 ± 10.6	**<0.001**
**Death ratio**	12 (10.9)	22 (40)	**<0.001**

**SVPS**: single ventriculoperitoneal shunt; **RVPS:** recurrent ventriculoperitoneal shunt; **CNS**: central nervous system; **NPH:** normal pressure hydrocephalus; **CSF**: cerebrospinal fluid. Data were expressed as mean ± SD.

**Table 2:** Diagnosis, clinical characteristics, and laboratory findings of patients.

	SVPS (n = 110)	RVPS (n = 55)	Normal Range Values	*p*
**Diagnosis**				**0.036**
Meningitis episodes	104 (94.5)	108 (72)		
Ventriculitis episodes	6 (6.5)	17 (18)		
**Clinical characteristics**				
Fever (≥38 C)	53 (44.9)	65 (55.1)		0.559
CNS infection findings	66 (46.2)	77 (53.8)		0.802
Intra-abdominal findings	11 (47.8)	12 (52.2)		0.918
Local infection findings	22 (55.0)	18 (45.0)		0.254
**Laboratory findings**				
White blood cell (10^9^/L)	12.8 ± 6.7	12.7 ± 6.3	4.0–11.0	0.511
C-reactive protein (mg/L)	14.8 ± 17.5	18.3 ± 27.3	0.0–3.0	**0.026**
Blood glucose (mg/dL)	102 ± 27	101 ± 30	70–100	0.832
CSF glucose (mg/dL)	31 ± 17	30 ± 15	50–75	0.073
CSF protein (mg/dL)	127 ± 79	185 ± 104	20–40	**<0.001**
Cell count of CSF (/mm^3^) (all mononuclear)	2114 ± 3556	2517 ± 4797	0.0–0.0	0.325

Data were expressed as mean ± SD. **SVPS:** single ventriculoperitoneal shunt; **RVPS:** recurrent ventriculoperitoneal shunt; **CNS:** central nervous system; **CSF**: cerebrospinal fluid.

**Table 3:** Grown microorganisms and resistance ratios in patients.

	SVPS (n = 110)	RVPS (n = 55)	*p*
**Type of grown pathogen**			**0.010**
Gram-positive bacteria	44 (60.3%)	33 (36.7%)	**0.004**
Gram-negative bacteria	28 (38.3%)	54 (60.0%)	**0.004**
*Candida* spp.	1 (1.4)	3 (3.3)	0.378
**Species**			
CoNS	34 (46.6)	18 (20.0)	
*S. aureus*	7 (9.6)	5 (5.6)	
*Streptococcus* spp.	1 (1.4)	4 (4.4)	
*Enterococcus* spp.	2 (2.7)	6 (6.7)	
*E. coli*	9 (12.3)	13 (14.4)	
*Pseudomonas* spp.	9 (12.3)	22 (24.4)	
*Klebsiella* spp.	7 (9.6)	11 (12.2)	
*Acinetobacter baumanni*	3 (4.1)	8 (8.9)	
**Resistance**			
*Methicillin in Staphylococci*	25/41 (61%)	20/23 (87%)	**0.029**
*3GCs*	17/28 (60.7%)	46/54 (85.2%)	**0.013**
*Piperacillin–Tazobactam*	12/28 (42.9%)	45/54 (83.3%)	**<0.001**
*Carbapenem*	4/28 (3.6%)	13/54 (10.4%)	**0.011**

**SVPS**: single ventriculoperitoneal shunt; **RVPS**: recurrent ventriculoperitoneal shunt; **CoNS**: coagulase-negative staphylococci; **3GCs**: third-generation cephalosporins; spp: species.

**Table 4:** Antimicrobial targeted treatment of patients.

	SVPS (n = 110)	RVPS (n = 55)	*p*
**Used Antibiotics**			
**Combinations without Carbapenem**	54 (51%)	20 (16%)	
3GCs–Glycopeptide	38	10	
Piperacillin–Tazobactam	5	4	
Piperacillin–Tazobactam–Glycopeptide	13	6	
**Combinations with Carbapenem**	54 (49%)	105 (84%)	**<0.001**
Carbapenem ± AG	18	19	
Carbapenem–Glycopeptide	19	52	
Carbapenem–Linezolid	8	11	
Carbapenem–Colimycin ± AG	8	16	
Carbapenem–Gylicopeptide–L-AmB	-	6	
Carbapenem–Linezolid–L-AmB	1	1	
**Duration of antibiotic use**	**21 ± 8**	**18 ± 6**	**0.003**
**Surgical Approach**			**0.001**
No surgery	22	36	0.118
Insertion after extraction	20	35	0.076
One-stage change	20	31	0.513
Two-stage change	44	21	**<0.001**
Extraction and no insertion	4	2	0.323

**SVPS:** single ventriculoperitoneal shunt; **RVPS**: recurrent ventriculoperitoneal shunt; **3GCs:** third-generation cephalosporins; **AG:** aminoglycoside; **L-AmB:** liposomal amphotericin B. Data were expressed as mean ± SD.

**Table 5:** Regression analysis of the occurrence of risk factors in patient group.

Risk Factors	OR (% 95 Confidence Interval)	*p*
**CSF leakage**	1.018 (1.004–1.033)	**0.012**
**History of previous shunt infections**	81.161 (17.843–369.179)	**0.000**
**CSF protein**	1.010 (1.005–1.015)	**0.000**
**Lack of 2-stage shunt exchange**	0.008 (0.000–0.378)	**0.014**
**CNS infection**	6.647 (1.512–29.226)	**0.012**

**CSF**: cerebrospinal fluid; **CNS:** central nervous system; **OR:** odds ratio.

## Data Availability

The original contributions presented in this study are included in this article. Further inquiries can be directed to the corresponding author.
